# Focal Hyperechoic Hepatic Lesions in Northern Region of Saudi Arabia: Prevalence, Radiologic Features, and Clinical Relevance

**DOI:** 10.3390/jcm14196987

**Published:** 2025-10-02

**Authors:** Fatimah M. Alonzi, Mohammed J. Alsaadi, Khaled Said Karam, Essa M. Alanzi, Noura K. Alhathal, Maram F. Alreshidi, Abdulrahman M. Alfuraih

**Affiliations:** 1 Radiology Department, King Khalid Hospital, Hail 55421, Saudi Arabia; fatimahalsahli1@gmail.com (F.M.A.); kkaram@moh.gov.sa (K.S.K.); ssmttr553@gmail.com (E.M.A.); 2 Radiology and Medical Imaging Department, College of Applied Medical Sciences, Prince Sattam Bin Abdulaziz University, Al-Kharj 11942, Saudi Arabia; 3 Radiology Department, Dr. Sulaiman Al Habib Hospital—Altakhasosi, Riyadh 13521, Saudi Arabia; nourahkhaaledt@gmail.com; 4 Radiology Department, Alshifa Hospital, Hail 55436, Saudi Arabia; mroomf1230@gmail.com

**Keywords:** hepatic lesions, computed tomography, ultrasound, tumours, hemangioma, adenoma, carcinoma

## Abstract

**Background:** This study investigates the incidence and radiological features of hyperechoic hepatic lesions in northern Saudi Arabia, using ultrasound (US) and computed tomography (CT). The aim is to evaluate the frequency of occurrence of these lesions and to describe the imaging characteristics of different focal hepatic lesions. **Methods:** A retrospective study was performed on 191 patients diagnosed with hyperechoic hepatic lesions at a single centre. Imaging data from ultrasound and CT scans were analyzed, including lesion number, segmental distribution, echogenicity, enhancement patterns, size, and type. Statistical methods included incidence calculation, variable correlation, and Pearson’s Chi-square test, with significance set at *p* < 0.05. **Results:** The incidence of hyperechoic hepatic lesions was 1.27%, with a higher prevalence in females (57.59%) and a median age of 40 years. Hemangiomas were the most common type of lesion (94.77%). Most lesions were solitary (87.43%) and benign (96.86%), with malignant lesions accounting for only 3.14%. A statistically significant age difference was observed between patients with benign and malignant lesions (*p* < 0.05). **Conclusions:** Focal hyperechoic hepatic lesions are relatively common in the northern Saudi population, with haemangiomas being the predominant benign entity. These lesions occur more frequently in women and are usually solitary. Ultrasound, complemented by problem-solving techniques such as contrast-enhanced ultrasound (CEUS) or triphasic CT, effectively characterizes hyperechoic hepatic lesions and guides clinical decisions regarding further evaluation or management.

## 1. Introduction

Liver disease is a leading global health burden, contributing substantially to morbidity and mortality, with prevalence driven by viral hepatitis, alcohol consumption, and, more recently, the epidemic of non-alcoholic fatty liver disease (NAFLD) [1,2]. Epidemiological patterns vary across regions, but in Saudi Arabia, the impact is amplified by high rates of obesity, diabetes, and metabolic syndrome, which collectively accelerate the progression of chronic liver disease [3,4,5]. These conditions predispose to a spectrum of hepatic abnormalities, ranging from benign to malignant lesions, and underscore the urgent need for reliable diagnostic strategies. The Kingdom has witnessed a rising incidence of chronic liver diseases, including hepatitis B and C infections, NAFLD, and hepatocellular carcinoma (HCC), with genetic, environmental, and lifestyle factors interacting to shape its unique epidemiological profile [4,5]. Benign hepatic lesions such as hemangiomas and focal nodular hyperplasia (FNH) coexist with malignant tumours including HCC and metastases, necessitating accurate imaging tools for timely differentiation and management. Hyperechoic hepatic lesions represent a heterogeneous group of focal abnormalities, varying from benign hemangiomas to malignant masses, making their precise characterization clinically relevant. Ultrasound remains the primary modality for evaluating hepatic lesions. Grayscale and Colour Doppler ultrasonography provide valuable diagnostic information, with characteristic findings such as the homogeneous hyperechogenicity of hemangiomas or the spoke-wheel vascular pattern of FNH [6]. However, diagnostic overlap between benign and malignant lesions persists, limiting the specificity of ultrasound alone [7]. To address this, multiphasic computed tomography (CT) and standardized reporting frameworks such as the Liver Imaging Reporting and Data System (LI-RADS) have been developed to improve reproducibility and diagnostic accuracy for hepatocellular carcinoma [8].

Additionally, contrast-enhanced ultrasound (CEUS) has emerged as a valuable adjunct, offering radiation-free, real-time characterization of indeterminate lesions. In this context, reliable epidemiological data on hyperechoic hepatic lesions in Saudi Arabia remain limited despite their growing clinical significance. Understanding their incidence, distribution, and imaging characteristics is critical for optimizing screening, improving early detection, and guiding resource allocation. The present study therefore aims to evaluate the prevalence and radiological features of focal hyperechoic hepatic lesions in the northern region of Saudi Arabia. Specifically, the study will compare the findings of ultrasound and CT in diagnosing focal hepatic masses, while recognizing that standardized reporting frameworks such as LI-RADS provide structured diagnostic criteria for CT/MRI characterization of hepatic lesions [8].

## 2. Materials and Methods

The study design was a retrospective and cross-sectional study. Patient data, including clinical notes, demographics, and ultrasound (US) and computed tomography (CT) studies, were retrieved from patient medical records and the standard picture archiving and communication system (PACS) at our centre in the North region of Saudi Arabia. The inclusion criteria included patients with focal hyperechoic hepatic lesions who had undergone abdominal and biliary US, as well as CT scans from March 2015 to March 2023. The exclusion criteria included all traumatic and cystic liver lesions. 

The patient consent form was waived due to the retrospective nature of this study, which was conducted in accordance with the Declaration of Helsinki. To demonstrate our commitment to ethical research, the ethics committee at our centre reviewed and approved all studies involving human participants (2024-19).

### 2.1. Scanning Parameters and Data Collection

All patients were scanned using the General Electric (GE) Logiq P5 (GE HealthCare, Waukesha, WI, USA) ultrasound machine, the Siemens Somatom Definition AS generation spiral CT (Siemens, Forchheim, Germany), and the GE Discovery CT (GE HealthCare, Waukesha, WI, USA). All patients were positioned in a supine position during scanning. Liver ultrasounds were performed with a convex broadband 3.5 MHz probe in various planes, including sagittal, parasagittal, transverse, oblique, subcostal, intercostal, and coronal planes. Contrast-enhanced ultrasound (CEUS) was not available in our centre during the study period. We acknowledge that CEUS is now a cornerstone in the characterization of focal liver lesions, and its absence represents a limitation of this study. A triphasic liver CT was performed with the following phases: arterial phase (25–35 s post-contrast injection), portal venous phase (60–70 s), and equilibrium/delayed phase (3–5 min). Serial CT slices were taken every 3 mm. Patients received an IV contrast dose of 1.5 mL/kg, with a total volume ranging from 80 to 120 mL, administered at a rate of 4–5 mL/s, as per departmental protocol. In some cases, preparation involved the administration of 1500–2000 mL of water or Gastrografin 30–60 min before the examination, serving as an oral contrast. The initial scan was a non-contrast scan. After administering oral and intravenous contrast, the liver was scanned during arterial imaging (early phase, 15 s), late phase (25–30 s), venous imaging (60 s), and equilibrium or excretory phase (3–4 min), with some cases delayed by 10–15 min for specific phases. Enhancement of each lesion during every phase was assessed and described based on the degree of hyperenhancement.

Additionally, non–ionic contrast agents, such as iohexol, were used. Consent was obtained and signed by both the patient and the referring physician. Contrast was administered via a peripheral IV. Continuous monitoring of vital parameters was maintained throughout contrast injection. The procedure details were explained to the patient. 

### 2.2. Study Population

A total of fifteen thousand one hundred and twenty-one (*n* = 15,121) US and CT scans were collected. After excluding cases without focal hepatic masses, 191 cases remained. One radiologist with over ten years of experience in the subspecialty of abdominal imaging reviewed the studies retrospectively. The diagnostic features and variables of hyperechoic hepatic lesions included in the study are as follows: number of lesions—single or multiple; location within the liver—lobar distribution (right lobe, left lobe, both lobes); ultrasound echogenicity (compared to normal liver parenchyma) and CT enhancement with contrast (hypodense and hyperdense); size and shape; margins of the lesion were classified as well-defined, poorly defined, regular, or irregular. The diagnostic criteria for lesion characterization were based on enhancement patterns consistent with contemporary standards, such as peripheral nodular enhancement with centripetal fill-in for hemangiomas, and arterial phase hyperenhancement with washout for HCC, consistent with LI-RADS guidelines [6,8].

### 2.3. Statistical Analysis

Data were analyzed using IBM SPSS Statistics, version 28 (IBM Corp., Armonk, NY, USA). Continuous variables (e.g., age and lesion size) were tested for normality using the Shapiro–Wilk test. Due to non-normal distributions, they were reported as medians with interquartile ranges (IQR). Categorical variables (e.g., gender, lesion number, type, location, malignancy status) were presented as counts and percentages. Non-parametric tests included the Mann–Whitney U test for comparing continuous variables between genders and the Kruskal–Wallis H test for assessing lesion size across lesion types. Pearson’s Chi-square test was used to evaluate associations between categorical variables. McNemar’s test compared the diagnostic accuracy of ultrasound and triphasic CT. Correlations were assessed using Spearman’s rank correlation for ordinal variables (e.g., lesion size, type) and point-biserial correlation for continuous variables with dichotomous outcomes (e.g., age vs. malignancy). Multivariable logistic regression identified predictors of malignancy, adjusting for age, gender, lesion size, and number. A *p*-value < 0.05 denotes statistical significance.

## 3. Results

This study analyzed the incidence, radiological characteristics, and diagnostic outcomes of focal hyperechoic hepatic lesions in 15,000 patients screened via ultrasound (US) at a hospital in northern Saudi Arabia. Of these, 191 patients with hyperechoic lesions were further evaluated using triphasic computed tomography (CT) for a definitive diagnosis.

### 3.1. Incidence of Focal Hyperechoic Hepatic Lesions

Among 15,000 screened patients, 191 had focal hyperechoic hepatic lesions detected on ultrasound (US), resulting in an incidence rate of 1.27% (95% confidence interval [CI]: 1.09–1.46%). All 191 lesions appeared hyperechoic on US and hypodense on computed tomography (CT). CT-based diagnoses revealed hemangiomas as the most common lesion (181 cases, 94.7%), followed by hepatocellular carcinoma (HCC; 4 cases, 2.2%), focal fatty infiltration (2 cases, 1.1%), and single cases (0.5% each) of metastasis, focal nodular hyperplasia (FNH), carcinoid tumour, and hamartoma. Benign lesions accounted for 97.4% (186 cases), while malignant lesions constituted 2.6% (5 cases). A Chi-square test indicated a significant association between lesion type and malignancy status (χ^2^ = 190.0, df = 6, *p* < 0.001) (Table 1).

### 3.2. Diagnostic Performance of Ultrasound and Triphasic CT

All lesions appeared hyperechoic on ultrasound and hypodense on computed tomography. Ultrasound accurately identified 181 cases (94.7%) as benign, misclassifying five cases as malignant (HCC and metastasis). Computed tomography achieved 97.4% accuracy (186/191), correctly identifying all benign cases and refining the diagnoses of malignant cases. A McNemar’s test indicated no statistically significant difference in diagnostic performance (*p* = 0.06), though CT demonstrated a numerically higher accuracy. (Table 2).

### 3.3. Lesion Characteristics and Demographic Correlations

**Figure 1:**
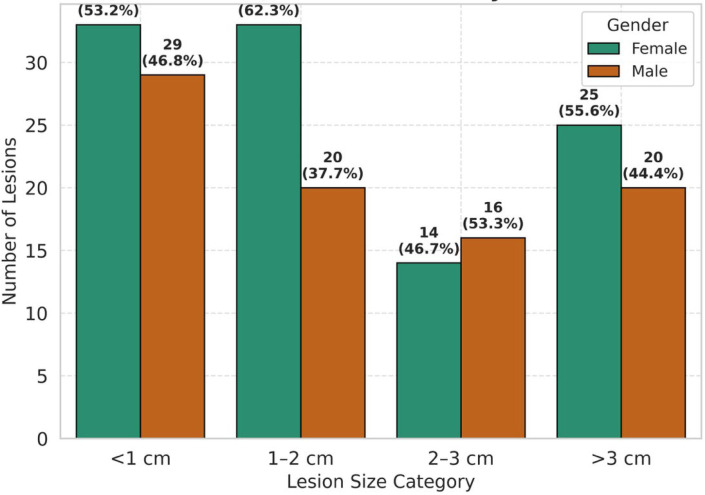
This grouped bar chart displays the number of lesions across four size categories (<1 cm, 1–2 cm, 2–3 cm, and >3 cm) stratified by gender. Counts and percentages are annotated above each bar. The figure shows that the distribution of lesion sizes is broadly similar between females and males, with no category showing a statistically significant difference (*p* > 0.05).

The cohort consisted of 106 females (55.3%) and 85 males (44.7%), with a mean age of 42.22 ± 16.5 years (range, 8–93 years). Lesions were predominantly single (174, 91.6%) and were most located in Segment 6 (35, 18.4%) or Segment 5 (33, 17.4%). The size distribution was as follows: <1 cm (62, 32.6%), 1–2 cm (53, 27.9%), 2–3 cm (30, 15.8%), and >3 cm (45, 23.7%). No significant differences were observed in lesion number (*p* = 0.42), size (*p* = 0.19), or malignancy status (*p* = 0.62) by gender (Chi-square tests). Correlations included: Age vs. Size: Spearman’s rho = 0.12, *p* = 0.10 (non-significant). Age vs. Number: Point-biserial r = 0.08, *p* = 0.27 (non-significant). Age vs. Malignancy: Point-biserial r = 0.15, *p* = 0.04 (weak positive correlation), as shown in Table 3, Figure 1.

### 3.4. Correlation Between Lesion Size and Type

**Figure 2:**
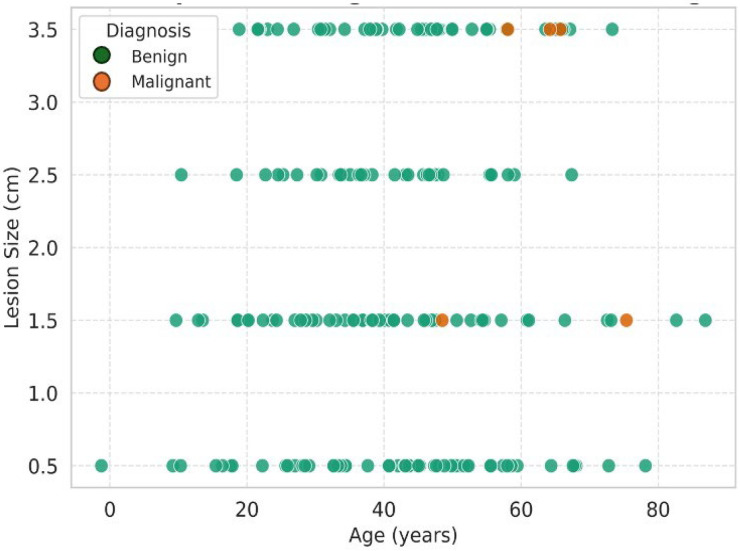
This scatter plot illustrates the relationship between patient age and lesion size, colour-coded by final diagnosis (benign vs. malignant). Solid circles improve clarity and visual distinction. Malignant lesions (in orange/orange) tend to cluster at larger sizes (>3 cm) and occur more frequently in older patients, supporting lesion size as a predictor of malignancy.

Lesion size varied significantly by type (Kruskal–Wallis H = 11.45, *p* = 0.04). Hemangiomas displayed a wide range of sizes, while malignant lesions (e.g., hepatocellular carcinoma, HCC) were mainly larger than 3 cm. By assigning ordinal codes (size: <1 cm = 1, >3 cm = 4; type: Hemangioma = 1, Hamartoma = 7), Spearman’s rho = 0.18 (*p* = 0.01) indicated a weak positive correlation between larger sizes and rarer, often malignant types, as shown in Table 4, Figure 2.

### 3.5. Clinical and Imaging Characteristics

Most lesions (149, 78.4%) were incidental findings, primarily detected during the evaluation for abdominal pain. All lesions appeared hyperechoic on ultrasound and hypodense on CT, with single lesions predominant (175, 91.6%) as shown in Table 5.

### 3.6. Predictors of Malignancy

**Figure 3:**
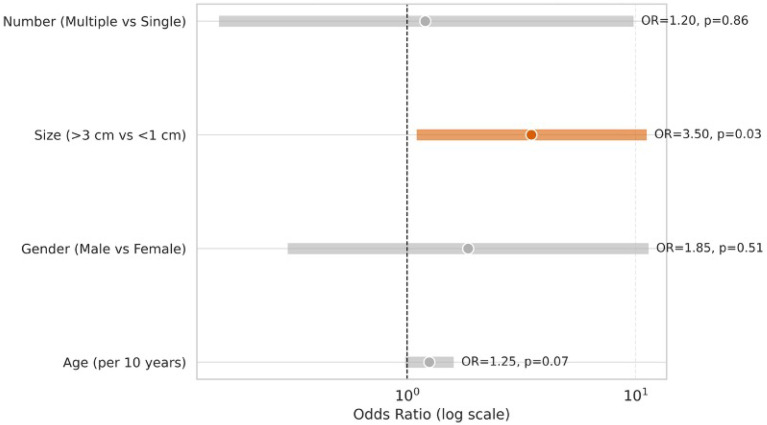
This forest plot presents odds ratios (OR) with 95% confidence intervals for potential predictors of malignancy: age, gender, lesion size, and lesion number. The *x*-axis uses a logarithmic scale. Size > 3 cm is the only statistically significant factor (OR = 3.50, *p* = 0.03), highlighted in orange/orange non-significant factors are shown in grey.

Multivariable logistic regression identified lesion size (>3 cm vs. <1 cm) as a significant predictor of malignancy (OR = 3.50, 95% CI: 1.10–11.2, *p* = 0.03). Age showed a non-significant trend (OR = 1.25 per 10 years, 95% CI: 0.98–1.60, *p* = 0.07). Gender and lesion number were not significant predictors, as shown in Table 6 and Figure 3.

## 4. Discussion

Focal hyperechoic liver lesions are commonly identified through pathological or imaging evaluations of the liver, encompassing a variety of malignant and benign neoplasms. Assessing these lesions is often a complex task and a primary focus of cross-sectional imaging studies. This study, utilizing retrospective data from ultrasound and CT images of focal hyperechoic hepatic lesions, aims to determine the incidence rate of these lesions in the northern region of Saudi Arabia and to discuss their radiological features. Notably, this study seeks to investigate the incidence rate of focal hepatic masses in the northern region of Saudi Arabia.

Our results demonstrated that hyperechoic liver lesions were overwhelmingly benign (97.4%), with hemangiomas comprising the vast majority (93.2%). Malignant lesions were rare (2.6%) and typically larger in size (>3 cm). These findings are strongly supported by the recent multicenter study [9], which showed that hyperechoic lesions ≤ 3 cm in patients without chronic liver disease or prior malignancy were uniformly benign and clinically insignificant. These findings suggest that small hyperechoic lesions in low-risk patients are generally clinically insignificant.

This study found that the incidence rate of focal hyperechoic hepatic lesions is approximately 1.27%. Our findings indicated that most focal hyperechoic liver masses were benign. Furthermore, hemangioma was identified as the most common type of benign hyperechoic hepatic lesion. Hepatocellular carcinoma (HCC) ranks as the second most common neoplasm and is the most prevalent primary liver malignancy.

In the current study, the standardized imaging protocol for detecting hepatic lesions involves an initial ultrasound followed by a triphasic spiral CT scan. The diagnostic characteristics of hepatic hemangiomas on ultrasound include well-circumscribed, well-defined, hyper-echoic appearances, often associated with distal acoustic enhancement. Small hemangiomas typically appear homogeneous, whereas larger hemangiomas (>4 cm) can display a heterogeneous appearance due to extra-lesion feeding vessels visible on Colour Doppler [10].

On non-enhanced CT scans, hemangiomas appear as well-defined, hypodense masses. During the arterial phase, they exhibit peripheral nodular enhancement, with progressive centripetal enhancement observed in the venous phase, and complete fill-in, appearing hyperattenuating to liver parenchyma, in the delayed phase. Atypical hemangiomas, in instances of complete thrombosis, demonstrate no enhancement. However, during the arterial phase, they may display diffuse enhancement (rapid fill-in) with a hyperdense appearance. In the delayed phase, they show incomplete filling in cases of partial thrombosis [11,12,13]. Hepatocellular adenoma (HCA) is an uncommon hepatic lesion associated with the use of oral contraceptives and anabolic steroids [13]. On ultrasound (US), HCA exhibits variable echogenicity (hyper-, iso-, or hypoechoic) due to fat, necrosis, or hemorrhage. Colour Doppler imaging may demonstrate prominent peripheral arteries and central draining veins within the lesion, improving diagnostic confidence [10,11,14,15,16]. On non-enhanced CT, HCA demonstrates variable attenuation (hyper-, hypo-attenuated). Contrast-enhanced CT typically reveals marked arterial-phase enhancement, followed by a rapid transition to iso- or hypodense attenuation relative to the hepatic parenchyma in the portal venous phase. In the delayed phase, HCA generally presents a homogeneous appearance, with hypoechoic areas indicative of hemorrhage, necrosis, or fibrosis [6,16,17].

Focal nodular hyperplasia (FNH), a rare finding among focal hyperechoic hepatic lesions in our study, is a benign liver lesion that can pose a significant diagnostic challenge when incidentally detected during abdominal imaging [7,10,15]. On ultrasound, FNH can appear as hyper-, iso-, or hypo-echoic. A characteristic feature of ultrasound is the ‘spoke-wheel’ pattern, which comprises central and radiating arterial vessels. In Colour Doppler imaging, these vessels often demonstrate a higher diastolic component compared to the systolic component. FNH typically exhibits a lobular contour, and power Doppler ultrasound can reveal blood flow within the central scar. This diagnostic challenge underscores the need for further research and a deeper understanding of FNH. On unenhanced and equilibrium-phase post-contrast CT, FNH appears iso-dense or minimally hypo-dense. It shows diffuse homogeneous substantial enhancement, except for the central scar or septa, which typically display minimal enhancement. During the venous phase, the lesion fades to iso-enhancement [10,11]. While FNH may be challenging to detect on baseline US and CT, CEUS has demonstrated high specificity by showing the characteristic spoke-wheel arterial pattern with persistent enhancement and absence of washout [7].

Fatty liver infiltration, a metabolic disorder, can lead to focal hepatic lesions detectable by ultrasound. Diffuse fatty infiltration increases liver echogenicity and attenuates the ultrasound beam. However, focal fatty infiltration and sparing can mimic neoplastic disease on ultrasound. These lesions appear hyper-echoic against the surrounding normal liver tissue. They are typically found in key areas, such as near the falciform ligament, the anteromedial portion of Segment IV, the hilar side of Segment IV, the anterolateral portion of Segment III, and the hepatic hilum. Notably, Colour Doppler imaging does not reveal any vascular abnormalities, providing reassurance about the absence of certain complications. On non-enhanced CT, these lesions appear iso-dense and maintain this iso-dense appearance through the arterial, venous, and delayed phases [18,19,20].

Bile duct hamartomas, congenital malformations of the ductal plate that do not connect to the bile ducts, are typically discovered incidentally during abdominal imaging. This underscores the importance of vigilance and thorough examination during routine imaging procedures. These lesions are usually small, ranging from 5 to 10 mm, and are widely distributed across all liver segments [21].

On ultrasound, they appear as small hyper-echoic or hypo-echoic lesions and may exhibit ringing artefacts, known as the comet tail appearance. On CT, bile duct hamartomas manifest as small cystic lesions with round, oval, or irregular shapes and do not show contrast enhancement. Recognizing the various forms of these lesions is crucial, as they may not always conform to a standard shape [9,18,22].

Hepatocellular carcinoma (HCC) primarily occurs in patients with chronic liver disease, particularly those with hepatitis B or C, liver cirrhosis, or haemochromatosis [10,17,20]. It is characterized by abnormal hepatocytes arranged in trabecular and sinusoidal patterns. HCC lesions may be solitary, multifocal, or diffusely infiltrating, showcasing variability in their characteristics. On ultrasound (USG), HCC lesions exhibit variable echogenicity—hyper-, hypo-, or iso-echoic. Increased echogenicity often results from intratumorally fat. Smaller lesions are typically homogeneous, whereas larger lesions are heterogeneous, adding to the complexity of HCC diagnosis. Colour Doppler imaging reveals intratumorally arterial vessels with irregular, tortuous tumour vessels, depending on the degree of differentiation [12]. A surrounding fibrous capsule is often present, appearing as a hypo-echoic rim encircling the lesion, which is characteristic of HCC [7,14,19]. Our HCC cases likely fulfilled LI-RADS 5 criteria on CT/MRI, which provides standardized diagnostic certainty for HCC [6,8]. On unenhanced CT images, most HCCs appear hypo- or iso-dense, particularly when they are small. Intratumoral fat can reduce CT attenuation and suggest the presence of primary HCC. During the arterial phase of contrast enhancement, HCCs enhance significantly, becoming iso- or hypo-dense in comparison to the liver parenchyma during the portal venous phase. Delayed-phase images typically display most HCC lesions as hypodense relative to the surrounding liver [19,20].

In a cohort of 228 hyperechoic liver lesions identified by ultrasound, only 6.1% were clinically actionable (requiring follow-up). Multivariable analysis identified cirrhosis (OR = 24.3), lesion size (OR = 1.77), and age (OR = 1.04) as the strongest predictors of clinical significance. Notably, hyperechoic lesions ≤ 3 cm in patients without a history of malignancy or underlying liver disease (including cirrhosis) were uniformly clinically insignificant (benign), suggesting that routine follow-up may be unwarranted in this low-risk subgroup [9]. Our study indicates similarly low rates of malignancy (2.6%, with 97.4% benign, predominantly hemangiomas at 93.2%), supporting the overall benign nature of these findings in screening contexts. Although our analysis did not assess cirrhosis or prior malignancy history, logistic regression confirmed lesion size as a significant predictor (OR = 3.50 for >3 cm vs. <1 cm, *p* = 0.03), with age approaching significance (OR = 1.25 per 10 years, *p* = 0.07). Consistent with the referenced findings, all lesions < 1 cm in our cohort were benign (*n* = 62), and malignancies were rare in smaller sizes (only 1 out of 145 lesions ≤ 3 cm), reinforcing size as a key risk factor and the potential to forgo routine follow-up for small lesions in low-risk patients.

### 4.1. Limitations

Hail City is one of the largest metropolitan areas in northern Saudi Arabia; however, despite its demographic and geographic importance, the study faces significant methodological limitations. A key concern is potential referral and selection bias, as not all cases of hyperechoic hepatic lesions in the northern region were recorded at our institution. This may have led to an underestimation of the true incidence, limiting the external validity and generalizability of the findings to the broader Saudi population or international groups.

Additional limitations should be acknowledged. Firstly, the single-centre, retrospective study design inherently predisposes to selection and information bias, while also limiting the robustness of causal inference. Secondly, histopathological confirmation was not systematically available for most lesions, with diagnostic classification mainly relying on imaging criteria and longitudinal follow-up. Although this reflects common clinical practice, it inevitably introduces some diagnostic uncertainty. Thirdly, the diagnostic algorithm did not include advanced modalities such as contrast-enhanced ultrasound (CEUS) or magnetic resonance imaging (MRI), both of which are increasingly regarded as reference standards for the characterization of focal hepatic lesions. Finally, the small number of malignant lesions (*n* = 5) significantly limits statistical power, precluding a definitive evaluation of risk factors predictive of malignancy.

### 4.2. Future Research Directions

Future research should focus on multicentre, prospective studies that include histopathological validation and standardized imaging frameworks (LI-RADS, CEUS). The use of advanced imaging techniques such as MRI with hepatobiliary agents, radiomics, and AI-based classification could further improve lesion characterization. Additionally, long-term studies in Saudi Arabia are necessary to monitor lesion outcomes and assess health system impacts, particularly in populations with high rates of NAFLD and metabolic syndrome.

## 5. Conclusions

Hyperechoic hepatic lesions are relatively frequent in the Saudi population, with the vast majority being benign, most commonly hemangiomas. Malignant lesions were rare, typically associated with larger size (>3 cm) and older age, highlighting lesion size as a significant predictor of malignancy. The observed incidence of focal hyperechoic hepatic lesions was approximately 1.25% among the studied cohort, with ultrasound and non-enhanced CT frequently detecting such lesions during routine abdominal imaging.

Our findings support the conservative management of small (<1 cm) hyperechoic lesions in low-risk patients, thereby reducing unnecessary follow-up. Triphasic CT demonstrated high diagnostic accuracy and should be considered a dependable problem-solving tool in cases where ultrasound findings are inconclusive. Furthermore, incorporating structured diagnostic frameworks such as LI-RADS, alongside advanced modalities including contrast-enhanced ultrasound (CEUS) and MRI, may further optimize clinical decision-making and minimize radiation exposure.

Future research should focus on multicenter, prospective studies integrating CEUS, MRI, and emerging AI-based imaging tools to refine risk stratification, improve diagnostic precision, and inform evidence-based guidelines tailored to the Saudi population and comparable international cohorts.

## Figures and Tables

**Table 1:** Distribution of Focal Hyperechoic Hepatic Lesions by Type, Number of Cases, Percentage, and Final Diagnosis (Benign/Malignant).

Lesion Type	Number of Cases	Percentage (%)	Final Diagnosis (Benign/Malignant)
Haemangioma	181	94.7	181 Benign
Hepatocellular Carcinoma	4	2.2	4 Malignant
Focal Fatty Infiltration	2	1.1	2 Benign
Metastasis	1	0.5	1 Malignant
Focal Nodular Hyperplasia	1	0.5	1 Benign
Carcinoid Tumour	1	0.5	1 Benign
Hamartoma	1	0.5	1 Benign
Total	191	100.0	186 Benign, 5 Malignant

**Table 2:** Ultrasound (US) and Computed Tomography (CT) Appearances and Diagnostic Accuracies for Focal Hyperechoic Hepatic Lesions.

Lesion Type	US Appearance	CT Appearance	Number of Cases	US Accuracy (%)	CT Accuracy (%)
Haemangioma	Hyperechoic	Hypodense	181	100	100
Hepatocellular Carcinoma	Hyperechoic	Hypodense	4	0	100
Focal Fatty Infiltration	Hyperechoic	Hypodense	2	100	100
Metastasis	Hyperechoic	Hypodense	1	0	100
Focal Nodular Hyperplasia	Hyperechoic	Hypodense	1	0	100
Carcinoid Tumour	Hyperechoic	Hypodense	1	100	100
Hamartoma	Hyperechoic	Hypodense	1	100	100
Total			191	94.7	97.4

**Table 3:** Comparison of Lesion Characteristics Between Female and Male Patients with *p*-Values from Chi-Square Tests.

Characteristic	Female (*n* = 106)	Male (*n* = 85)	*p*-Value (Chi-Square)
Number of Lesions			0.42
Single	99 (93.4%)	76 (89.4%)	
Multiple	7 (6.7%)	9 (10.6%)	
Size			0.19
<1 cm	34 (31.5%)	29 (34.1%)	
1–2 cm	33 (31.4%)	20 (23.5%)	
2–3 cm	14 (13.3%)	16 (18.8%)	
>3 cm	25 (23.8%)	20 (23.5%)	
Diagnosis			0.62
Benign	104 (98.2%)	82 (96.5%)	
Malignant	2 (1.9%)	3 (3.5%)	

**Table 4:** Distribution of Focal Hyperechoic Hepatic Lesions by Type and Size Categories.

Lesion Type	<1 cm	1–2 cm	2–3 cm	>3 cm	Total
Hemangioma	63	52	27	36	178
Hepatocellular Carcinoma	0	1	0	3	4
Focal Fatty Infiltration	0	1	1	0	2
Metastasis	0	0	0	1	1
Focal Nodular Hyperplasia	0	0	0	1	1
Carcinoid Tumour	0	0	0	1	1
Hamartoma	0	1	0	0	1
Total	63	53	30	45	191

**Table 5:** Summary of Demographic and Clinical Features of Patients with Focal Hyperechoic Hepatic Lesions.

Factor/Feature	Value/Description
Total Patients	191
Gender	Female: 106 (55.3%), Male: 85 (44.7%)
Age (Mean ± SD)	42.22 ± 16.5 years
Age Range	8–93 years
Reason for Referral	Incidental (abdominal pain): 149 (78.4%), Other: 42 (21.6%)
US Appearance	Hyperechoic: 191 (100%)
CT Appearance	Hypodense: 191 (100%)
Number of Lesions	Single: 175 (91.6%), Multiple: 16 (8.4%)
Size Distribution	<1 cm: 62 (32.6%), 1–2 cm: 53 (27.9%), 2–3 cm: 30 (15.8%), >3 cm: 45 (23.7%)
Most Common Location	Segment 6: 35 (18.4%)
Final Diagnosis (CT)	Benign: 185 (97.4%), Malignant: 5 (2.6%)

**Table 6:** Logistic Regression Analysis of Factors Associated with Malignancy in Focal Hyperechoic Hepatic Lesions.

Feature	Odds Ratio (OR)	95% CI	*p*-Value
Age (per 10 years)	1.25	0.98–1.60	0.07
Gender (Male vs. Female)	1.85	0.30–11.4	0.51
Size (>3 cm vs. <1 cm)	3.50	1.10–11.2	0.03
Number (Multiple vs. Single)	1.20	0.15–9.80	0.86

## Data Availability

The data presented in this study are available within the article.

## Abbreviations

The following abbreviations are used in this manuscript:

NAFLD	Non-alcoholic fatty liver disease
HCC	Hepatocellular carcinoma
US	Ultrasound
CT	computed tomography
PACS	Picture archiving and communication system
GE	General Electric
FNH	Focal Nodular Hyperplasia
HCA	Hepatocellular Adenoma
CEUS	Contrast-Enhanced Ultrasound
LI-RADS	Liver Imaging Reporting and Data System
